# A Chinese multicenter retrospective study of isolated increased nuchal translucency associated chromosome anomaly and prenatal diagnostic suggestions

**DOI:** 10.1038/s41598-021-85108-6

**Published:** 2021-03-10

**Authors:** Hua Jin, Juan Wang, Guoying Zhang, Hongyan Jiao, Jiansheng Zhu, Zhimin Li, Chen Chen, XuanPing Zhang, Huan Huang, JiaYin Wang

**Affiliations:** 1 Department of Prenatal Diagnosis, Ji’nan Maternal and Child Health Care Hospital, Ji’nan, Shandong China; 2 School of Computer Science and Technology, Xi’an Jiaotong University, Xi’an, Shaanxi China; 3 Department of Obstetrics and Gynecology, The First Affiliated Hospital of Nanjing Medical University, Nanjing, Jiangsu China; 4 Department of The Branch Center of Prenatal Diagnosis, Shijiazhuang Maternal and Child Care Service Hospital, Shijiazhuang, Hebei China; 5 Medical Genetic Center, Maternity and Child Health Hospital of Anhui Province, Affiliated Maternity and Child Health Hospital of Anhui Medical University, Anhui, China; 6 Annoroad Gene Technology (Beijing) Co., Ltd, Beijing, China; 7 Zhejiang Annoroad Biotechnology Co., Ltd, Zhejiang, China

**Keywords:** Genetic testing, Genetic testing

## Abstract

Extensive researches involving fetuses with multiple ultrasound anomalies have been conducted over the years, but only few were focused on the isolated increased nuchal translucency (NT). On top of that, these limited number of researches were all designed as single-arm studies and the control group was missing. In this study, we conducted a multicenter, retrospective study using amniotic fluid samples collected from 1197 pregnant women having fetuses with isolated increased NT (INT group) or normal NT values (NNT group). Copy number variation sequencing (CNV-seq) was performed to determine their chromosome status and pathogenic variations were validated using SNP array. Overall, 59 chromosome aneuploidies, 34 pathogenic CNVs and 23 copy number variants of unknown significance (VOUS CNVs) were discovered. the INT group had a significantly higher proportion of aneuploidy (19.44%) and pathogenic CNV (8.33%) than the control group (3.49% and 2.30% respectively), and 88.89% of the pathogenic CNVs were related to heart defects. Additionally, more male fetuses were presented in the INT group (68.51%), but they did not have a higher risk (Relative Risk = 1.03) of carrying pathogenic chromosome variations than female fetuses. Our results demonstrated that fetuses with isolated increased NT had a distinct pattern of chromosome abnormality and majority of detected pathogenic CNVs could be linked to the congenital heart disease. Furthermore, because a considerable proportion of pathogenic CNVs were detected, we strongly recommend to perform a joint test of karyotyping and CNV analysis in prenatal diagnosis for fetuses with isolated increased NT in order to decrease the incident of missed diagnosis.

## Introduction

For several decades, ultrasonography was widely used in prenatal diagnosis as a first-tier screening method and the thickness of nuchal translucency (NT) measured between 11^+0^ and 13^+6^ weeks in the first trimester was considered as an important indicator for Down syndrome^1,2^. In the clinical management guideline published by The American College of Obstetricians and Gynecologists (ACMG) and Society for Maternal Fetal Medicine, an increased NT is determined when the thickness of nuchal translucency is more than 3.0 mm or above the 99th percentile of the crown-rump length. The risk of trisomy 21 can be elevated to 1 in 46 when NT > 99th, whereas the risk was 1 in 8806 when NT < 95th^3^. Hence, it is recommended that karyotyping should be conducted to confirm the chromosome status of fetuses with increased NT. Moreover, number of previous researches have shown that copy number variation (CNV) could also be detected in fetuses with increased NT^4–9^ and the cause of increase by pathogenic CNVs (pCNVs) was through blood and lymph circulation disruption^10^.

Fetus with increased NT is a frequently observed symptom. While majority studies primarily concentrated on fetuses with multiple ultrasound indications, very few were focused on the ones with isolated increased NT. Previous publications demonstrated that on top of the increased incidents of chromosome aneuploidies. various percentages of pathogenic CNVs ranging from 0% to 12.8%^11–13^ and high prevalence of congenital heart defects (CHD) and neurodevelopmental disorders were could also be found in fetuses with isolated increased NT^14^. Unfortunately, these researches were all designed as a single-arm study, and without a proper control group, results can only be compared with external historical data of other related work, which made it difficult to diminish the effects caused by different enrollment criteria among studies. In this work, we conducted a multicenter retrospective study with a control group to investigate chromosomal characteristics of fetuses with isolated increased NT and next-generation sequencing (NGS) based CNV-seq method was chosen to identify chromosome abnormalities because of its high sensitivity and specificity^15,16^. Our results showed that 8.33% of the fetuses in the isolated increased NT group carried pCNVs, which was significantly different (*P* < 0.001, relative risk = 3.62) than those in the normal NT group. Additionally, discrepancy in the distribution of pCNVs across chromosomes between the two groups was also observed and the risk of CHD was higher in fetuses with isolated increased NT. Furthermore, majority of cases in the isolated NT group were male (68.5%, 74/108), but their relative risk of having chromosome anomalies was the same (relative risk = 1.03) as female fetuses of the same group.

## Results

### Sample information

In this study, we analyzed 1197 amniotic fluid samples of high-risk Chinese pregnant women with singleton pregnancy. Samples were successfully tested using next-generation sequencing platform and they were all free of maternal contamination. Fetuses with normal NT and without any other ultrasound soft marker abnormalities was assigned as the normal NT group (NNT), whereas the ones with isolated increased NT was stratified as the isolated increased NT group (INT). The NNT group contained 1089 samples, the INT group had 108 cases and the mean maternal age was 32.43 ± 5.71 years and 30.19 ± 4.89 years respectively.

### Detection of chromosomal abnormalities

Overall, a total of 116 anomalies was detected among 1197 samples, 59 (4.93%) of which had chromosome aneuploidies, 34 (2.84%) carried pathogenic CNVs and 23 (1.95%) had copy number variants of unknown significance (VOUS CNVs). The INT group contained 21 (19.44%, 21/108) aneuploidy cases, 9 (8.33%, 9/108) pCNVs, 1 (0.93%, 1/108) VOUS CNVs and 77 (71.29%, 77/108) normal cases, whereas the ratio was 3.49% (38/1089), 2.30% (25/1089), 2.02% (22/1089) and 92.19% (1004/1089) in the NNT group respectively (Table 1). In comparison with the NNT group, the ratio of fetuses with normal karyotype, chromosomal aneuploidy and pCNV was significantly different in the INT group (all *P* < 0.001) and the relative risk of normal, aneuploidy and pCNV was 0.77, 5.57 and 3.62 respectively. The ratio of cases with VOUS CNVs, however, was not significantly different between the two groups (*P* = 0.429).

**Table 1:** Incidence of aneuploidy and CNV in NNT and INT groups.

Anomalies	NNT Group (N = 1089)		INT Group (N = 108)		RR^†^	*P*
	N	%	N	%		
Normal	1004	92.19	77	71.30	0.77	< 0.001***
Aneuploidy	38	3.49	21	19.44	5.57	< 0.001***
Pathogenic CNV	25	2.30	9	8.33	3.62	< 0.001***
VOUS CNV	22	2.02	1	1.23	0.61	0.429

Abbreviations: NNT: normal nuchal translucency; INT: increased nuchal translucency; CNV, copy-number variation; VOUS, variants of unknown significance; RR, relative risk.

^†^Relative risk is calculated as % cases of the anomaly in the INT Group/% cases of the corresponding anomaly in the NNT Group, e.g. RR of aneuploidy: 19.44%/3.49% = 5.57. The NNT group is used as the reference.

***P < 0.001.

### Comparison of chromosomal variations between NNT and INT group

**Figure 1:**
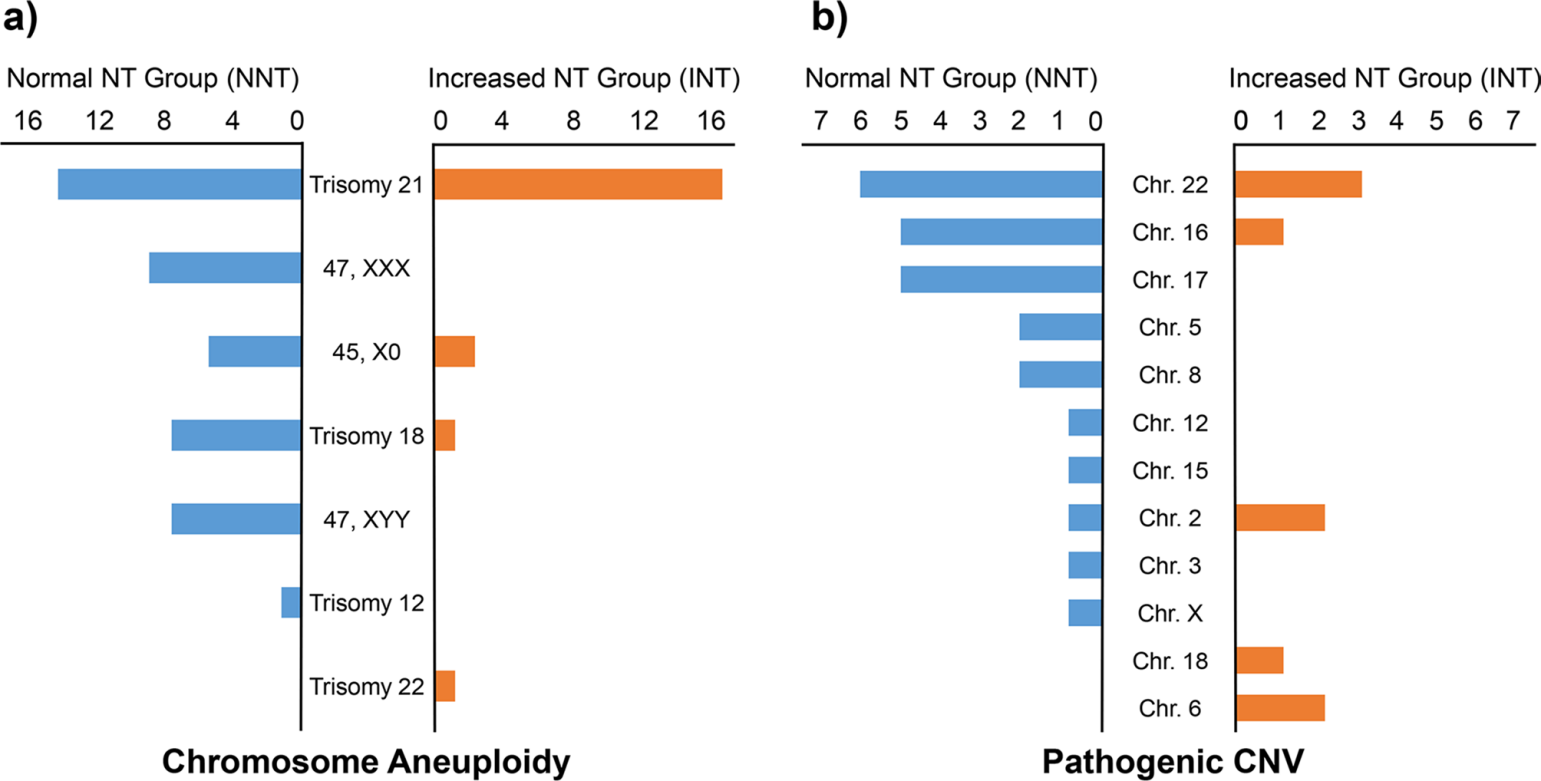
Distribution of chromosome aneuplodies and pathogenic CNVs in NNT and INT groups. (**a**) Number of chromosome aneuploidies showed distinct distribution patterns in NNT (blue) and INT groups (orange); (**b**) Number of pathogenic CNVs revealed that distribution of CNVs was very different between NNT (blue) and INT (orange) groups.

The 108 samples in the INT group, can be further divided into two sub-groups on the basis of NT thickness (Table 2). In the 3–4 mm NT sub-group, the percentage of chromosomal aneuploidy, pathogenic CNV and VOUS CNV cases was 14.81% (12/81), 7.41% (6/81) and 1.23% (1/81) respectively and it was 33.33% (9/27), 11.11% (3/27) and 0.00% (0/27) in the NT > 4 mm sub-group. Statistical analysis did not show any significant difference in the rate of pathogenic CNV and VOUS CNV between the two groups (*P* = 0.546, 0.562 respectively). The *P* value of chromosome aneuploidy was 0.035, demonstrating a sign of increase with increased NT thickness. Moreover, the distribution of aneuploidy in the INT group was also different to that of the NNT group (Fig. 1a). In the INT group, trisomy 21 occupied 80.95% (17/21) of the aneuploidies and the rest of 4 cases were X0 (2/21, 9.52%), trisomy 18 (1/21, 4.76%) and trisomy 22 (1/21, 4.76%). The anomalies in the NNT group, on the other hand, were spread out between trisomy 21 (14/38, 36.84%), trisomy X (8/38, 21.05%), trisomy 18 (5/38, 13.16%), X0 (5/38, 13.16%), XYY (5/38, 13.16%) and trisomy 12 (1/38, 2.63%).

**Table 2:** Incidence of aneuploidy and CNV discovered in NT 3–4 mm and > 4 mm sub-groups.

Anomalies	NT 3–4 mm (N = 81)		NT > 4 mm (N = 27)		RR^†^	*P*
	N	%	N	%		
Normal	62	76.54	15	55.56	0.73	0.037*
Aneuploidy	12	14.81	9	33.33	2.25	0.035*
Pathogenic CNV	6	7.41	3	11.11	1.50	0.546
VOUS CNV	1	1.24	0	0	0	0.562

Abbreviations: NT: nuchal translucency; CNV, copy-number variation; VOUS, variants of unknown significance; RR, relative risk.

^†^Relative risk is calculated as % cases of the anomaly in the NT > 4 mm group/% cases of the corresponding anomaly in the NT 3–4 mm group, e.g. RR of aneuploidy: 33.33%/14.81% = 2.25. NT 3–4 mm group is used as the reference.

* P < 0.05.

**Figure 2:**
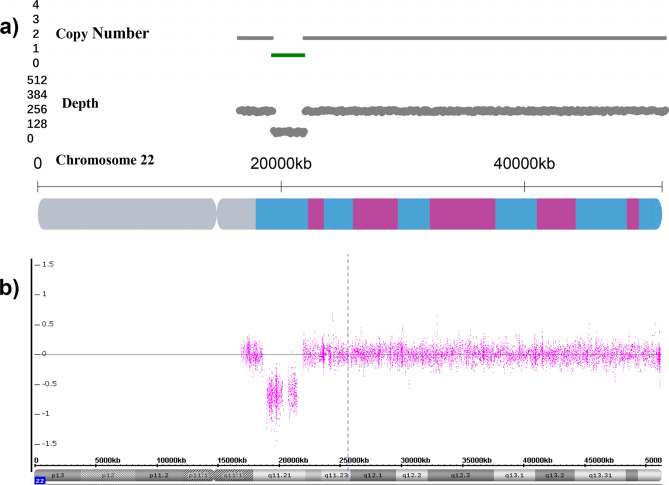
CNV-seq analysis and SNP array confirmation of the fetus with Di George syndrome (sample ID: 00936). (**a**) CNV‐seq analysis showed the presence of 22q11 deletion (green) in the amniotic fluid sample; (**b**) SNP array validated the 22q11 deletion in the fetus.

In terms of pathogenic CNVs, a total of 25 pCNVs in the NNT group and 9 pCNVs in the INT group were discovered (Table 3). One example of the pCNV detected by CNV-seq and SNP array were illustrated in Fig. 2. In the NNT group (Fig. 1b), 64% of the pathogenic CNVs were located on chromosome 16 (5/25, 20.00%), chromosome 17 (5/25, 20.00%) and chromosome 22 (7/25, 28.00%). The top 3 frequently observed pCNVs were 22q11 duplication syndrome (5/25, 20.00%), 16p11.2 duplication syndrome (3/25, 12.00%) and Charcot-Marie-Tooth syndrome type 1A (2/25, 8.00%). In comparison, 77.78% of pCNVs in the INT group were located (Fig. 1b) on chromosome 2 (2/9, 22.22%), 16 (2/9, 22.22%) and 22 (3/9, 33.33%), and 22q11 deletion syndrome (also known as the DiGeorge syndrome) had the highest occurrence rate (3/9, 33.33%).

**Table 3:** The list of pathogenic CNVs identified in the normal NT and enlarged NT groups.

Group	Sample ID	Fetus sex	CNV position^†^	Size (Mb)	Chromosomal disorder^‡^	Source of evidence	Reported cases with CHD and neurodevelopmental disorders^§^
NNT	00433	F	chr2:g.10001_24135000dup	24.13	Partial trisomy 2p	Lurie et al.^43^	CHD, ID
NNT	00376	F	chr3:g.196965295_197415294dup	0.50	3q29 duplication syndrome	#611936	ID
NNT	00745	F	chr4:g.10001_4015500del	4.00	Wolf-Hirschhorn syndrome	#194190	CHD, ID
NNT	00806	M	chr5:g.10001_11160000del	11.15	Cri du Chat Syndrome	#123450	ID, rare CHD
NNT	00117	F	chr5:g.60001_6560000del	6.50	Cri du Chat Syndrome	#123450	ID, rare CHD
NNT	00925	M	chr8:g.12510001_23560000dup	11.05	8p23.1 duplication syndrome	ORPHA:251076	CHD, ID
NNT	00324	M	chr12:g.60001_34810001dup	34.75	Trisomy 12p	ORPHA:1699	CHD, ID
NNT	00823	M	chr15:g.31976624_32526623del	0.55	15q13.3 deletion syndrome	#612001	ID, rare CHD
NNT	01229	M	chr16:g.15460001_16310000dup	0.85	16p13.11 microduplication syndrome	ORPHA:261243	ID, rare CHD
NNT	00271	M	chr16:g.25160001_26560000dup	1.40	16p11.2p12.2 microduplication syndrome	ORPHA:261204	rare ID, rare Schizophrenia
NNT	00702	F	chr16:g.29460001_30210000dup	0.75	16p11.2 duplication syndrome	#614671	ID; Schizophrenia
NNT	00692	F	chr16:g.29610001_30210000dup	0.60	16p11.2 duplication syndrome	#614671	ID, Schizophrenia
NNT	00319	M	chr16:g.29610001_30160000del	0.60	16p11.2 deletion syndrome	#611913	ID, ASD
NNT	00234	M	chr17:g.1_25400000dup	25.40	Trisomy 17p	ORPHA:261290	ID, rare CHD
NNT	00300	M	chr17:g.14050001_15500000dup	1.45	Charcot-Marie-Tooth syndrome type 1A	#118220	HMSNs
NNT	00457	M	chr17:g.14100001_15500000dup	1.40	Charcot-Marie-Tooth syndrome type 1A	#118220	HMSNs
NNT	01424	M	chr17:g.16250001_22750000dup	6.50	Potocki-Lupski syndrome	#610883	Autism, CHD, ID
NNT	01532	M	chr17:g.34800001_36250000del	1.45	Renal cysts and diabetes syndrome	#137920	rare ID
NNT	00837	F	chr22:g.18600001_21900004dup	3.30	22q11 duplication syndrome	#608363	ID, rare CHD
NNT	01069	F	chr22:g.18900001_21500004dup	2.60	22q11 duplication syndrome	#608363	ID, rare CHD
NNT	00593	M	chr22:g.18900001_25150004dup	6.25	22q11 duplication syndrome	#608363	ID, rare CHD
NNT	00317	F	chr22:g.18950001_2145000dup	2.50	22q11 duplication syndrome	#608363	ID, rare CHD
NNT	00952	F	chr22:g.20700005_21500004dup	0.80	22q11 duplication syndrome	#608363	ID, rare CHD
NNT	00042	F	chr22:g.18850001_21600004del	2.75	DiGeorge syndrome	#188400	ADHD, CHD, rare Autism
NNT	00395	M	chr22:g.45700005_51150004del	5.45	Phelan-McDermid syndrome	#606232	rare ID
INT	10003	M	chr2:g.61087852_61537851del	0.45	2p16.1-p15 deletion syndrome	#612513	ADHD, Autism, ID
INT	00608	M	chr2:g.178879852_184979851del	6.09	2q31.2 deletion syndrome	#612345	ID, rare CHD
INT	00178	M	chr6:g.88610001_100310000del	11.7	Interstitial 6q microdeletion syndrome	Vignoli et al.^44^	Autism, CHD, ID
INT	00305	F	chr6:g.128060001_171010000dup	42.95	Partial duplication of 6q	Conrad et al.^45^	CHD, ID
INT	00523	F	chr16:g.14860001_16660000dup	1.80	16p13.11 microduplication syndrome	ORPHA:261243	ADHD, CHD, ID
INT	00218	F	chr18:g.41460001_77210000dup	35.75	Partial trisomy 18q	Cereda et al.^46^	CHD. ID
INT	00136	F	chr22:g.18900001_21700004del	2.80	DiGeorge syndrome	#188400	ADHD, CHD, rare Autism
INT	00936	M	chr22:g.18900001_21500004del	2.60	DiGeorge syndrome	#188400	ADHD, CHD, rare Autism
INT	00451	M	chr22:g.18800001_21700004del	2.90	DiGeorge syndrome	#188400	ADHD, CHD, rare Autism

Abbreviations: NNT, normal NT; INT, increased NT; M, male; F, female; CNV, copy-number variation; ORPHA, Orphanet database; ADHD, attention deficit hyperactivity disorder; ASD, autism spectrum disorder; CHD, congenital heart disease; HMSNs, hereditary motor and sensory neuropathies; ID, intellectual disability.

^†^The positions of CNV are written in accordance with the International System for Human Cytogenomic Nomenclature (ISCN, 2016).

^‡^The name of the disorder is written as the entry name of OMIM (Online Mendelian Inheritance in Man) database, Orphanet database or descriptions in the cited articles.

^§^Symptom description of the corresponding pathogenic CNV was obtained from OMIM database, Orphanet database or cited articles.

## Discussion

Increased NT detected by ultrasonography at 11^+0^ to 13^+6^ weeks is considered to be an effective indicator for fetal anomalies. Currently, it is used to predict up to 50% of major fetal abnormalities^20^ and it was associate with high levels of several adverse outcomes including miscarriage, fetal anomalies and genetic syndromes^21–24^. The mechanism of nuchal elevation is diversified. Cystic hygroma is a type of manifestation characterized by fluid-filled cavities (cysts) and it was found to be associated with trisomy 13, trisomy 18 and Turner's syndrome (45, X0). The nuchal elevation exsited in non-45,X fetuses with cystic hygroma was caused by the proliferation of lymphatic vessels^25,26^. In Turner's syndrome, however, the primary cause of cystic hygroma is local aplasia of lymphatic vessels, which interrupts the drainage into the jugular lymph sacs^27^. By contrast, nuchal oedemas, a second type of NT elevation appearing as a non-echogenic area of over 3.5 mm below the skin and outside the cervical vertebral column in ultrasonic scans^28^, is closely associated with trisomy 21. High level of precipitate around bundles of collagen fibrils and over-expression of collagen type VI in nuchal skin of fetuses could be observed^28,29^. Significant increase of hyaluronan was also found in fetuses with trisomy 21, suggesting that it may be involved in the pathogenesis of increased NT^30^.

Different from previous researches, we focused on the fetuses with isolated increased NT in this work. By doing so, we could then investigate their molecular characteristics and provide a strong basis for guiding the choice of subsequent diagnostic strategies in clinical settings. Overall, we analyzed and compared chromosomeal variations between NNT control group (1089 samples) and INT group (108 samples). For chromosome aneuploidy, previous researches demonstrated that the rate of aneuploidy was positively correlated with increased NT thickness. The percentage of chromosomal defects was 3.7% when NT value was between 2.5–3.5 mm and it was increased to 21.1% or above 60% when the NT thickness reached 3.5–4.5 mm or over 6.5 mm respectively^12^. In our work, we also demonstrated a significant increase in the percentage of aneuploidies and the incident rate was increased from 14.81% to 33.33% in NT 3.0–4.0 mm and NT > 4.0 mm subgroups. Moreover, the differences in the distribution of aneuploidy between the NNT and INT groups suggested that the increased NT value was closely associated with trisomy 21, whereas aneuploidies of 47, XXX and 47, XYY were less likely to cause the elevation.

Unlike chromosome aneuploidy, CNVs were frequently found in human genomes^31^ and based on its influence to human health, it is classified as benign, VOUS and pathogenic. Since the clinical significance of VOUS CNVs is not clear, we only focused on assessing the relationship between pathogenic CNVs in this work. The potential biological effects of pathogenic CNVs on the relevant diseases were believed to be related to the deletion or duplication of morbid genes. For example, Kerzendorfer et al.^32^ Discovered that deletion of *SLBP* or *NELFA* genes may contribute to the clinical features of Wolf-Hirschhorn syndrome, such as growth retardation and microcephaly. Both of these genes are involved in histone biogenesis and all patient cell lines with variable deletion showed delayed progression from S-phase to M-phase of the cell cycle and defective DNA replication and reduced levels of chromatin-associated histones after DNA replication were also observed. Medina et al.^33^ Demonstrated that the *CTNND2* gene was closely related to the mental retardation phenotype of cri-du-chat syndrome by showing the strong correlation between hemizygous loss of *CTNND2* and severe mental retardation. CTNND2 is a neuronal specific protein and expressed during early development and involved in cell motility. These properties may support its role in the mental retardation of the syndrome when presented in only one copy. Moreover, micro RNA (MIR) also seem to be associated with CNV related disease. Weber et al. Closely examined a case of 8p23.1 duplication syndrome through SNP array analysis, he found that dosage sensitive genes such as *SOX7*, *TNKS1*, *MIR124-1* and *MIR598* were located in the core duplicated interval. Both *MIR124-1* and *MIR598* genes have been implicated in neuropsychiatric disorders and so he suggested that these two MIRs might be a contributing factor to autism spectrum disorder in the 8p23.1 duplication syndrome patient^34^. Finding the morbids genes, however, is a difficult and ongoing task because the deleted or duplicated region may contain many genes. For example, 3q29 duplication syndrome is a CNV related disease, most patients have eye abnormalities, intellectual disability and small head. It has an additional copy of 1.6 Mb at position 29 on chromosome 3 and this duplicated segment contains about 20 genes. Although some of these genes are thought to be involved in brain and eye development, it is still unknown which specific genes, when abnormally copied, are related to the varied signs and symptoms of the syndrome.

In this work, our data showed that the percentage of pCNVs detected in fetuses in the INT group was 8.33%, which was consistent to the previously reported range of 6.86% to 9.09% in NT > 99th samples^12,35^, and it was significantly higher than that of the NNT group. However, no significant difference in pCNV incident rate was found between 3.0–4.0 mm and > 4 mm sub-groups and this suggested that, unlike chromosome aneuploidy, the occurrence of pathogenic CNVs would not increase further when NT value was above 3 mm. In addition, previous literatures reported that fetus with increased NT may have an increased risk of CHD^4^ and different hypotheses on the aetiology of the increased NT have been suggested^36^. Since our enrollment criteria excluded fetuses with other ultrasound anomalies including heart defects, we therefore looked into potential symptoms which could be caused by the identified pathogenic CNVs. The description of symptoms were collected from OMIM (Online Mendelian Inheritance in Man) and ORPHANET database, whereas the rest of information was obtained from appropriate literatures.

In our data, the most common pathogenic CNVs detected in the INT group was Di George syndrome, which was well-known to cause malformation of the heart. Other pCNVs, such as chromosome 2q31.2 deletion syndrome, 6q microdeletion syndrome, partial 6q duplication, 16p13.11 microduplication syndrome and partial trisomy 18q, of the same group were also reported to cause heart defects with the exception of chromosome 2p16.1-p15 deletion syndrome (1/9, 11.11%). In contrast, only 9 out of 25 pathogenic CNVs in the NNT group, namely partial trisomy 2p, Wolf-Hirschhorn syndrome, 8p23.1 duplication syndrome, trisomy 12p, chromosome 15q13.3 deletion syndrome, 16p13.11 microduplication syndrome, Trisomy 17p, Potocki-Lupski syndrome and Di George syndrome, could be linked to venticular septal defect, atrial septal defect or hypoplastic left heart, whereas the rest of 16 (64%) pCNVs have not yet established a clear association to CHD. What’s more, difference in types of pCNVs was also discovered between the two groups, 68.00% (17/25) of pCNVs in the NNT group had microduplications, whereas 66.67% (6/9) of pCNVs in the INT group were microdeletions. However, due to the limited number of samples, this finding needs to be further validated using larger cohort and its potential implications also need to be investigated. Furthermore, it was reported that the gender of fetus may also play a role in the increased NT thickness^37^ and we have investigated this claim in our work. The results showed that 68.51% (74/108) of the INT group were male fetuses and 31.48% (34/108) were female fetuses which was in concordance with the previous finding. However, the ratio of pathogenic chromosome anomalies in males and females was almost identical (*P* = 0.94), 24.32% (18/74) and 23.53% (8/34) respectively. Male fetuses did not have higher risk (RR = 1.03, female is used as the reference) of carrying pathogenic chromosome variations than female fetuses.

Despite our best efforts to distinguish nuchal hygroma from nuchal oedemas using ultrasonography, we could not completely rule out the possible inclusion of nuchal hygroma samples, which is a limitation of this study. Although the cases of trisomy 18 and Tuner sydrome in the INT group showed no signs of cysts under ultrasound examination, miscarriage tissues were not collected and examined and so the exact nature of the NT elevation was undetermined. Also, this work only focused on the effects of chromosomal aneuploidy and copy number variation on the increased NT, which was mainly caused by incorrect seperation of chromosome or chromosome lost.

during the maturation process of germ cells or early cleavage of fertilized eggs and non-allelic homologous recombination respectively. Environmental factors such as malnutrition may also associate with heart or neural abnormal development, but it is not examined in this work. According to previous literatures, prenatal malnutrition might have an impact on genetic selection, which would increase the chance of obtaining de novo genetic mutations in germ cells and transmit to the offspring if the starvation occurs before fertilization or elevate the probability of passing on the alleles of neuropsychiatric disorders to the next generation when pregnant women under nutritional stress^38,39^. In addition, transcriptome analysis using mouse model under prenatal nutritional deficient environment revealed altered gene expression profile and discovered 15 key genes related to autism and schizophrenia^40–42^.

In summary, NT thickness was originally proposed to evaluate the risk of Down syndrome and fetuses with isolated increased NT anomaly are usually diagnosed with the karyotyping alone, but we demonstrated in this work that fetuses with isolated increased NT also had additional 8.33% of pathogenic CNVs on top of chromosome aneuploidies, and 88.89% of which appeared to be associated with heart defects. Although no signs of CHD were discovered by the ultrasound throughout the entire pregnancy, these fetuses with pCNVs would be highly likely to develop severe symptoms during infancy or childhood. Therefore, it is most beneficial to integrate CNV analysis and karyotyping in prenatal diagnosis to avoid missed diagnosis.

## Material and methods

### Sample collection

This was a joint retrospective study with prenatal diagnosis centers across four Chinese provinces Anhui, Jiangsu, Shandong and Shannxi. The enrollment criteria was: 1. women underwent amniocentesis procedure between 16 + 0 to 18 + 6 weeks; 2. singleton pregnancy; 3. pregnant women without inheritable risk, tumor, pre-eclampsia, and prior risk of abnormal pregnancy outcome; 4. crown-rump length was between 45 and 84 mm; 5. NT was measured between 11 + 0 and 13 + 6 weeks; 6. fetuses without any ultrasound anomalies (NNT group); 7. fetuses with increased NT (INT group) and no other ultrasound soft marker abnormalities (e.g. nuchal hygroma, mild ventricular expansion, intracardiac echogenic focus, hyperechogenic bowel, absence or dysplasia of nasal bone, short long bones, single umbilical artery, choroid plexus cyst, increased cisterna magna and hydronephrosis etc.); 8. CNV-seq analysis was carried out using amniotic fluids; 9. pathogenic CNVs detected by CNV-seq were confirmed by CMA.

In total, 1197 pregnant women between January 2017 and March 2019 were enrolled, their archived clinical records and sequencing results were retrieved from the Jinan Maternal and Child Health Care Hospital, the First Affiliated Hospital of Nanjing Medical University, the Shijiazhuang Maternal and Child Care Service Hospital and the Maternity and Child Health Hospital of Anhui Province, Affiliated Maternity and Child Health Hospital of Anhui Medical University. Pregnant women were stratified into 2 categories on the basis of the NT thickness, the normal NT group (NNT group) and the isolated increased NT group (INT group). The NNT group consists of fetuses with the NT value less than 3 mm. The INT group, on the other hand, contained fetuses with NT ≥ 3 mm.

For 59 pregnant women with chromosome aneuploidies, 9 of which experienced miscarriage, 43 had induced abortion, and 7 with fetuses carrying 47, XYY or 47, XXX continued their pregnancy and gave birth. Six pregnant women with cases of DiGeorge syndrome and Cri du Chat Syndrome chose to take induced abortion, whereas the rest of CNV cases continued their pregnancy and gave birth to a living infant.

### CNV-seq

CNV-seq was performed by following manufacture’s protocol. In short. total genomic DNA was isolated from amniotic fluid samples using the Amp Genomic DNA Kit (TIANGEN Biotech, Beijing, China) according to the user’s manual. Next generation sequencing was performed as previously described^17^. In short, genomic DNA was fragmented to the average size of 200 bp and 2.5 ng of fragmented DNA was used for the sequencing library construction. Barcoded sequencing adaptors were ligated to the DNA fragments and amplified by the polymerase chain reaction (PCR). The PCR product was then purified using magnetic beads and the constructed libraries were pooled and sequenced with NextSeq 550AR platform (Annoroad Technology, China). Finally, 8–10 million of 35 bp single-end raw reads were generated for each sample.

After sequencing quality control and trimming, short reads were aligned to the human reference genome (hg19) with the BWA aligner. Unique reads were counted for each of the 100 kb window. GC bias of per window read counts was corrected using the LOWESS model. The normal-karyotype database (NKD), derived from a group of 1000 samples with normal karyotype confirmed by G-banded karyotype analysis, was used as the background. Algorithms used for the bioinformatics analysis was detailed in the previous literature^18^. According to the recommendations of the ACMG standards and guidelines^19^, CNVs detected by the analysis were classified as pathogenic, variants of unknown significance (VOUS) or benign. Benign CNVs was usually considered as population polymorphism, thus samples with benign CNVs were classified as normal in this study.

### CMA validation

SNP array was used in all four institutes to verify the pathogenic CNVs identified by the CNV-seq analysis. Briefly, Affymetrix Genechip CytoScan 750 K SNP Array (ThermoFisher, Shanghai, China) was used according to the user’s manual for the CNV identification with the average inter probe distance of 100 kb. The accompanied data analysis software Chromosome Analysis Suite (ChAS, version 1.2.1, ThermoFisher, https://www.thermofisher.com/cn/zh/home/life-science/microarray-analysis/microarray-analysis-instruments-software-services/microarray-analysis-software/chromosome-analysis-suite.html) was used to calculate the log2 intensity ratio.

### Statistical analysis

The differences between groups were examined using the chi-square test by Statistical Product and Service Solutions software (SPSS, version 22, IBM, https://www.ibm.com/analytics/spss-statistics-software). A significant P value was defined as 0.05.

### Ethical approval

This study was approved by the institutional ethics committee of each hospital, including the Jinan Maternal and Child Health Care Hospital, the First Affiliated Hospital of Nanjing Medical University, the Shijiazhuang Maternal and Child Care Service Hospital and the Maternity and Child Health Hospital of Anhui Province, Affiliated Maternity and Child Health Hospital of Anhui Medical University, with the exemption of patient’s informed consent because only retrospective data was used. All experiments were performed in accordance with relevant regulations and details.

## Data availability and material

Sequence data of this study are available upon reasonable request.
